# Poly(Ethylene Oxide)-based Electrolyte for Solid-State-Lithium-Batteries with High Voltage Positive Electrodes: Evaluating the Role of Electrolyte Oxidation in Rapid Cell Failure

**DOI:** 10.1038/s41598-020-61373-9

**Published:** 2020-03-09

**Authors:** Gerrit Homann, Lukas Stolz, Jijeesh Nair, Isidora Cekic Laskovic, Martin Winter, Johannes Kasnatscheew

**Affiliations:** 1 Helmholtz-Institute Münster, IEK-12, Forschungszentrum Jülich GmbH, Corrensstraße 46, 48149 Münster, Germany; 2 MEET Battery Research Center, Institute of Physical Chemistry, University of Münster, Corrensstraße 46, 48149 Münster, Germany

**Keywords:** Energy science and technology, Energy storage, Batteries

## Abstract

Polyethylene oxide (PEO)-based solid polymer electrolytes (SPEs) typically reveal a sudden failure in Li metal cells particularly with high energy density/voltage positive electrodes, *e.g*. LiNi_0.6_Mn_0.2_Co_0.2_O_2_ (NMC622), which is visible in an arbitrary, time – and voltage independent, “voltage noise” during charge. A relation with SPE oxidation was evaluated, for validity reasons on different active materials in potentiodynamic and galvanostatic experiments. The results indicate an exponential current increase and a potential plateau at 4.6 V vs. Li|Li^+^, respectively, demonstrating that the main oxidation onset of the SPE is above the used working potential of NMC622 being < 4.3 V vs. Li|Li^+^. Obviously, the SPE│NMC622 interface is unlikely to be the primary source of the observed sudden failure indicated by the “voltage noise”. Instead, our experiments indicate that the Li | SPE interface, and in particular, Li dendrite formation and penetration through the SPE membrane is the main source. This could be simply proven by increasing the SPE membrane thickness or by exchanging the Li metal negative electrode by graphite, which both revealed “voltage noise”-free operation. The effect of membrane thickness is also valid with LiFePO_4_ electrodes. In summary, it is the cell set-up (PEO thickness, negative electrode), which is crucial for the voltage-noise associated failure, and counterintuitively not a high potential of the positive electrode.

## Introduction

State-of-the-art (SOTA) Li ion batteries (LIBs) include a liquid electrolyte composed of LiPF_6_ salt in a solvent mixture of ethylene carbonate combined with at least one linear carbonate *e.g*. dimethyl carbonate, diethyl carbonate or ethylmethyl carbonate^1,2^.

The use of metallic Li instead of insertion anodes is considered for enhancement of energy density^3,4^. Given safety issues (*e.g*. by flammability of liquid electrolytes) and insufficient compatibility with Li metal based negative electrodes, the actual research trend is re-discovering solid electrolytes, *e.g*. inorganic (ceramic or glassy) electrolytes and organic solid polymer electrolytes (SPEs) again^5–7^.

Compared to inorganic electrolytes, SPEs may offer the chance for easier processing with composite electrodes, which may result in better wettability and more conformal interfaces, thus less contact resistances^5,8^, which is supposed to be more important for overall kinetics than bulk ion conduction^1^. The SOTA SPE, often used as benchmark, is based on polyethylene oxide (PEO), which is recognized as the polymer for solid electrolytes in lithium based batteries since the 1970s^9,10^. The physicochemical properties of PEO based electrolytes have been thoroughly investigated over the past decades^11–15^. For example, it is known that despite the low dielectric constant (ε_r_ ≈ 5) the dissociation of the Li salt proceeds via chelating complexation of the oxygen entities in PEO, which is energetically favorable compared to its chemical relatives *e.g*. polymethylene oxide or polypropylene oxide^16^. Moreover, Li^+^ conduction could be assigned to the segmental chain motion in the amorphous phases (via “free volume” domains), thus PEO displays Vogel-Fulcher-Tamman (VFT) behavior (in contrast to the Arrhenius behavior of Li^+^ transport in ceramic/glassy solid or many liquid electrolyte media)^12,17^. While amorphous phases are beneficial for Li^+^ conduction, they decrease the mechanical stability, hence creating a trade-off in properties. A lower glass transition temperature (T_g_) is regarded as an indication for a higher amount of amorphous phases^17^. The T_g_ (and related ionic conductivity) can be tailored for example via the molecular weight (MW) of PEO, the Li salt concentration^18,19^, incorporation of nano-particles^20^, cross linking^21^, or plasticizers^22–24^.

Still, the reproducibility and predictability of T_g_ or ionic conductivity is challenging because of the complexity of PEO based systems on microscopic level, as for example, usage and storage conditions can sensitively impact the content of possible phases, thus the accompanied properties significantly. Also, the assumed ability for the suppression of high surface area lithium (HSAL)^25,26^, in the most detrimental morphology of dendrites and the associated compatibility with Li metal^6^ is debated because from a physical point of view a shear modulus above 6 GPa is required, which is hard to achieve for PEO above T_g_^27,28^.

There is also a lack of knowledge from the point of view of battery cell application, especially for the reactivity and compatibility at the positive electrode side^17^. Even though better electrochemical stability is speculated compared to liquid electrolytes, because of lower reactivity of macromolecules and accompanied lower rate of convection^1,17^, it is believed that decent cycle life can only be achieved for low redox potential positive electrodes due to the low oxidative stability of ether groups in PEO (≈4.0 V vs. Li|Li^+^)^1,29^. For determination of oxidative stability of electrolytes, potentiodynamic approaches on inactive electrodes are typically used, where the practical validity of obtained conclusions is in question^30,31^.

In this work, we look at the PEO based SPE from the point of view of battery cell operation in order to understand cell failure and to unravel reported ambiguities in regard to oxidation stability. A diagnostic analysis of the origin of the well-known “voltage noise” failure in Li metal based batteries with high energy/high voltage electrodes, *e.g*. LiNi_0.6_Mn_0.2_Co_0.2_O_2_ (NMC622), is of particular interest and both interfaces, the SPE|Li and the SPE|NMC622 will be regarded.

## Experimental

### Materials

Poly(ethylene oxide) (PEO, M_w_ 100.000 Da to 5.000.000 Da) and 1-Methyl-2-pyrrolidinone (NMP, anhydrous, 99.5%) were purchased from Sigma-Aldrich. Lithium bis(trifluoromethanesulfonyl)imide (LiTFSI, 99.9%) and Polyvinylidene difluoride (PVdF, Solef 5130) were purchased from Solvay, France. Super C65 carbon black was received from Imerys, France. Mylar foil (100 µm thickness) was purchased from DuPont, USA. Battery grade electrolyte, 1 M LiPF_6_ in a mixture of ethylene carbonate and ethyl methyl carbonate (EC/EMC 3:7 by wt.) (LP57 Selectilyte) from BASF, was used as benchmark liquid electrolyte. The active materials LiNi_0.6_Mn_0.2_Co_0.2_O_2_ (NMC 622), LiNi_0.5_Mn_1.5_O_4_ (LNMO), LiMn_2_O_4_ (LMO) and LiFePO_4_ (LFP) were purchased from Targray, Canada. Graphite ready-to-use negative electrodes were provided by Customcells, Germany. Lithium metal (Abermale) was used as counter and reference electrode. All electrodes had a diameter of 12 mm, except Pt with 1 mm. Material storage and sample preparations was performed in a dry-room (dew point −65 °C). PEO was dried under vacuum (10^−7^ mbar) at 45 °C and LiTFSI at 110 °C for 2 days before use. All other chemicals were used as received.

### PEO membrane preparation

Free-standing PEO-LiTFSI polymer membranes were prepared by mixing of PEO and LiTFSI in a mortar using different EO:Li ratios. The mixture was sealed in a coffee bag and stored in an oven at 60 °C for 2 days to improve the homogeneous dissolution of LiTFSI in PEO. Similar to literature^32^, the resulting gum-like material was sandwiched between Mylar foil sheets and pressed for 10 min at 100 °C with an applied pressure of 15 bar, yielding a pinhole free membrane. The thickness of the resulting membrane in the range of 100 ± 5 µm was controlled by usage of a spacer (Mylar foil). The molecular weights of PEO and respective EO:Li ratios (20:1, 15:1, 12:1) are mentioned in the corresponding sections. If not mentioned, PEO with 300.000 Da and an EO:Li ratio of 12:1 is used.

### Electrode preparation and cell assembly

NMC 622 electrodes consisting of 91 wt% NMC 622, 4 wt% Carbon Black and 5 wt% PVdF were prepared by dissolving PVdF in NMP followed by the addition of carbon black and NMC622. The mixture was homogenized using a dissolver. The slurry was casted on aluminium foil using a doctor blade with a wet coating thickness of 50 µm. The electrode sheets were dried for 3 hours at 80 °C under vacuum, punched into circular electrode and dried again over night at 120 °C before use. The average active mass loading of NMC622 electrodes was 4.1 mg cm^−2^. For the LNMO electrodes 84 wt% LNMO, 8 wt% Carbon Black and 8 wt% PVdF were used. For the LMO electrodes 80 wt% LMO, 10 wt% Carbon Black and 10 wt% PVdF were used. For the LiFePO_4_ (LFP) electrodes 90 wt% LMO, 5 wt% Carbon Black and 5 wt% PVdF were used. The LNMO, LMO and LFP electrodes were prepared using the procedure described above. The average active mass loading was 6.3 mg cm^−2^, 3.2 mg cm^−2^, 7.2 mg cm^−2^, respectively. Slight differences in composition and mass loading of the electrodes have no significant impact as long as mild kinetic conditions are used, as applied in this work, which is low rate (15 mA g^1^ ≈ 0.1 C) and elevated temperatures (60 °C)^33–35^. All cells used in galvanostatic cycling tests were prepared in two electrode setup (coin cell) using the above mentioned positive electrodes as working electrode, PEO-LiTFSI as polymer membrane and lithium metal or graphite as negative electrode. Cells used for the determination of the oxidative stability were prepared in three-electrode setup using the above mentioned positive electrodes as working electrode, and lithium metal as counter and reference electrode^36^. Stated membrane thicknesses refer to the thickness before cell assembly at room temperature.

### Electrochemical measurements

All constant current cycling and potentiodynamic experiments were conducted on a Maccor Series 4000 battery cell test system and VMP device at 60 °C in a climate chamber (Binder KB400). The used experimental conditions are mentioned within the respective text and/or figure captions.

## Results and discussion

### Failure during charge: Impact of PEO oxidation?

**Figure 1:**
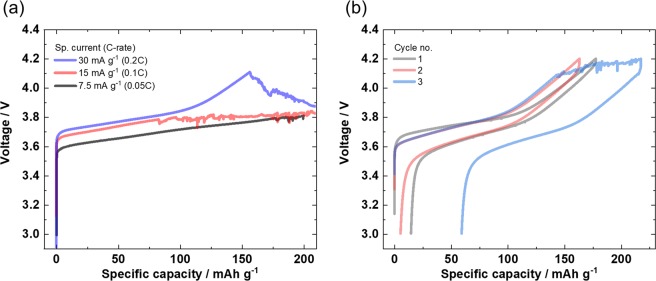
Galvanostatic charge/discharge cycling of a PEO based SPE in a NMC622||Li cell within a voltage range of 4.2–3.0 V demonstrating the difficulties in prediction and reproduction. (**a**) With varied specific currents (C-rates). (**b**) “Lucky shot” for a cell using a specific current of 30 mA g^−1^, where the failure (noisy voltage response during charge) occurred in the third cycle.

The galvanostatic operation of a NMC622||Li cell with PEO based SPE reveals a typical noisy voltage charge curve. As shown in Fig. 1(a) for varied specific currents (or C-rates), this failure is independent of time or voltage, thus demonstrating its arbitrary nature, where the electrochemical behavior is rather unpredictable and irreproducible.

However, some cells frequently reveal complete noisy-free charge/discharge cycles (“lucky shots”) with a characteristic NMC622 voltage profile^35,37^, which is shown in Fig. 1(b) for the initial two charge/discharge cycles. In this example, the typical failure during charge occurs only in the third cycle. Interestingly, the subsequent discharge proceeds without failure, having a reasonable specific discharge capacity of 159 mAh g^−1^. The untypical specific charge capacity (218 mAh g^−1^) in the previous (third) cycle hints to a contribution of side reactions contributing to charge capacity, which is gained during the failure processes in the noisy voltage range. Apparently, PEO based SPE principally can work without this voltage noise even in a NMC622||Li cell (cycle no. 1 and 2 in Fig. 1b), thus for cell voltages above 4 V and can reveal reasonable capacity values contrary to frequent beliefs^1,29^. Though increased internal resistances are obvious (*e.g*. increased *IR*-Drop) between cycle no. 1 and no. 2, these cycles are cycle-able without abnormal characteristics (“voltage noise”). A thorough investigation of the performance including internal resistance is beyond the scope of this work and part of upcoming works.

**Figure 2:**
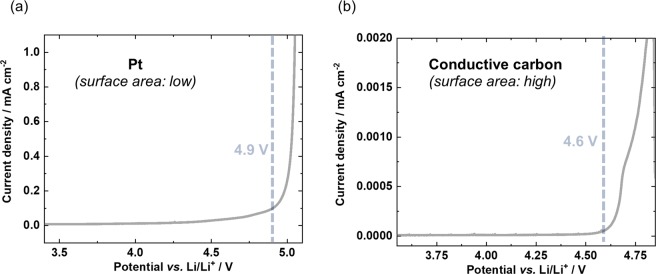
Potentiodynamic approach using LSVs of PEO based SPE with a scan rate of 0.1 mV s^−1^ on (**a**) Pt and (**b**) conductive carbon working electrode resulting in an exponential increase in current density of 4.9 and 4.6 V vs. Li/Li^+^, respectively. Higher oxidation onset of Pt can be attributed to higher overpotentials because of significantly lower surface area, thus higher current densities^30^.

Given the fact that voltage noise failure proceeds only during charge and results in additional/parasitic capacity, oxidative instability of the PEO based SPE may be the cause. For validation reasons the determination of the main oxidation onset is experimentally investigated using both, the conventional potentiodynamic approach via linear sweep voltammetry (LSV) and the galvanostatic approach^30,31^. The onset of oxidation can be detected in each approach by an exponential (Butler-Volmer like) current increase and a potential plateau, respectively^30,31^. A LSV based on typically used (inert) Pt working electrode is depicted in Fig. 2(a). While low currents are observed already at potentials above 4.0 V vs. Li|Li^+^, an exponential increase in current density at ≈ 4.9 V vs. Li|Li^+^, points to the main oxidation onset of the PEO based SPE. However, the validity of this result is questionable, as the surface area of Pt foil is small compared to composite battery electrodes in practical cells^38^ and furthermore the electro-catalytic activity of Pt may be different. Overpotentials associated with the high current density and decreased “surface activity” may lead to misleading values^30^. To verify this relation, LSV based on conductive carbon (higher surface area)^39^ is applied (Fig. 2b), which reveals an obvious lower onset potential (4.6 V vs. Li/Li^+^). This electrolyte oxidation reaction may also be in part accompanied by anion intercalation into conductive carbon^40^.

**Figure 3:**
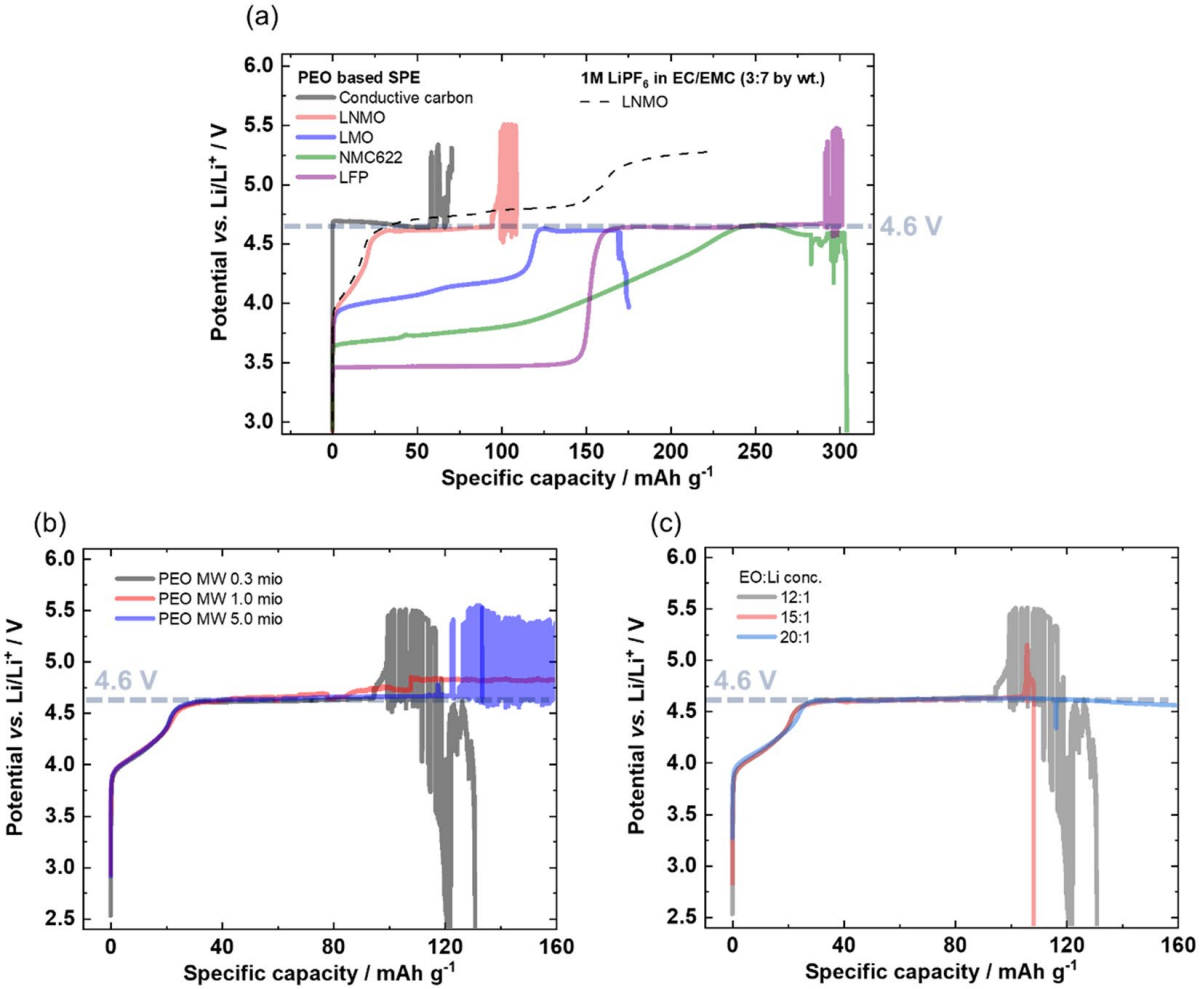
Determination of the onset of main oxidation of PEO based SPEs via overcharge of the working electrode with a specific current of 15 mA g^−1^. (**a**) Using different positive electrodes: LMO, LNMO, NMC622, LFP and conductive carbon. Independent of the positive electrode (structure, chemical composition) similar potential plateaus can be observed, demonstrating its main oxidative stability limit at 4.6 V vs. Li|Li^+^. (**b**) The dependence on molecular weight (MW) of PEO on the oxidation onset is checked with an LNMO electrode. No dependence on MW can be concluded as confirmed by similar potential plateaus at 4.6 V vs. Li|Li^+^. (**c**) The dependence on salt concentration in PEO on the oxidation onset is checked on LNMO electrode, as well. No dependence can be concluded as confirmed by similar potential plateaus at 4.6 V vs. Li|Li^+^.

Despite higher surface area, the validity can be still questioned because of a significant difference to practical electrodes and their “interface”^30^. Therefore, a galvanostatic approach is carried out on practically relevant composite electrodes based on NMC622 as well as on Ni-containing and Ni-free spinel type active materials, LiNi_0.5_Mn_1.5_O_4_ (LNMO) and LiMn_2_O_4_ (LMO), respectively, also on the olivine structure LiFePO_4_ (LFP) and conductive carbon^31^. The electrodes are galvanostatically charged with a specific current of 15 mA g^−1^ as shown in Fig. 3(a). Similar to the potentiodynamic approach, also the galvanostatic approach reveal 4.6 V vs. Li/Li^+^ as oxidation onset, as seen by the respective potential plateau. The LMO electrodes show their typical delithiation reaction during charge in the potential range of 3.9–4.2 V vs. Li|Li^+^ ^41,42^. The subsequent potential plateau at 4.6 V vs. Li|Li^+^ points to the onset of main oxidation of the electrolyte (parasitic reaction^43^). This potential plateau can also be seen on the LNMO electrode. Contrary to LMO, the apparent continuous oxidative decomposition reaction of the PEO-based SPE, which remains at 4.6 V vs. Li|Li^+^, prevents the characteristic delithiation reaction of LNMO in the potential range of 4.7–4.8 V vs. Li|Li^+^, while it can be achieved with conventional liquid electrolytes, *e.g*. LiPF_6_ in EC/EMC (3:7 by wt.)^31^. Finally, the same electrolyte oxidation potential plateau is observed on NMC622 and LFP, above their charge process characteristic for the respective delithiation reaction^43,44^. The obtained equal potential plateaus, being independent of the electrochemical determination method, the structure and the composition of the respective positive electrode material, point at the main oxidation reaction of the PEO based SPE. Contrary to the results obtained from the potentiodynamic method on Pt, the determination on high surface area electrodes reveals an oxidative stability of the bulk electrolyte up to 4.6 V vs. Li|Li^+^ at 60 °C. Though, small, but possible oxidation processes in the long term e.g. during continuous charge/discharge cycling are not excluded, which may contribute to capacity loss and capacity fade, it is for sure, that the main electrolyte oxidation is happening at this potential^45^.

In a further experiment possible relation between the chain length/molecular weight (MW) of the deployed PEO and the decomposition onset potential, which has been assumed in literature^17^, is checked in this work. The electrochemical stability measurements on LNMO of PEOs with different MWs (0.3 m, 1 m, 5 m) and Li salt concentrations (EO:Li 20:1, 15:1, 12:1) for PEO are shown in Fig. 3(b,c), respectively. For all investigated MWs and Li salt concentrations the charge process resulted in a potential plateau at 4.6 V vs. Li|Li^+^, which points at the main oxidative limit of the different PEO based SPEs. It can be concluded, that the main oxidative decomposition onset of the SPE membranes is apparently independent of MW- and Li salt concentration under the used conditions.

Electrochemical bulk oxidation of the PEO-based SPE can be excluded as reason for voltage noise failure, as the respective noise in the charge curve is also detected at potentials below the PEO oxidation onset potential (Fig. 1).

### Impact of the use of Li metal as anode

**Scheme 1:**
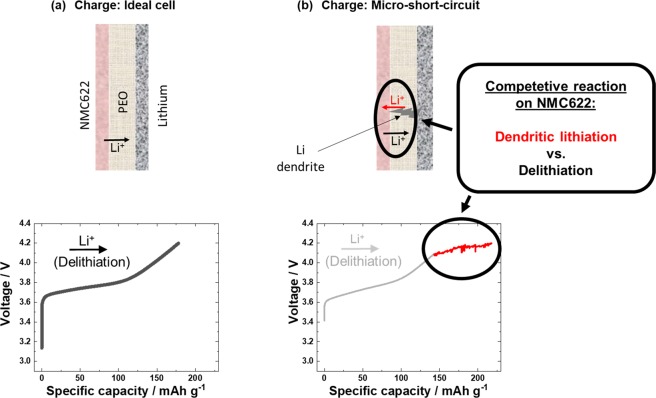
Charge process for a NMC622||Li cell with PEO based SPE (**a**) for an ideal cell: The delithiation results in the characteristic voltage increase. (**b**) For a cell with micro-shorts: The conventional delithiation of NMC622 is counter-acted by dendritic lithiation caused by a Li dendrite. This competitive reaction can lead to an arbitrary voltage increase/decrease resulting the observed “voltage noise”.

From experience of lab and commercial cell behavior, irreproducible, high and abnormal deviations in the specific charge capacities during cycling, particularly during C-rate tests, may be attributed to the Li metal negative electrode (in three electrode arrangement: Li as counter electrode)^36^. An indication is *e.g*. visible by high surface area lithium (HSAL)^25,26^ deposits, such as dendrites, on the Li metal side of the separator after cell disassembly. These dendrites may penetrate through the separator and form micro shorts that can result in a temporary chemical (dendritic) lithiation (=re-discharge) of NMC622, which actually can occur in parallel to the electrochemical delithiation (=charge) process. As schematically shown in Scheme 1, the possible competitive reaction between dendritic lithiation and NMC622 delithiation may result in fluctuation (arbitrary increase and decrease) of voltage/potential, resulting in the “noisy” response as seen in Figs. 1 and 2.

As a separation of the sticky PEO based SPE membrane from the negative and positive electrodes (a common challenge of *post mortem* analysis of solid state batteries), is experimentally challenging, alternative experiments are conducted to verify a relation between Li dendrite penetration and failure of NMC622|SPE|.Li cells.

### SPE membrane thickness

**Figure 4:**
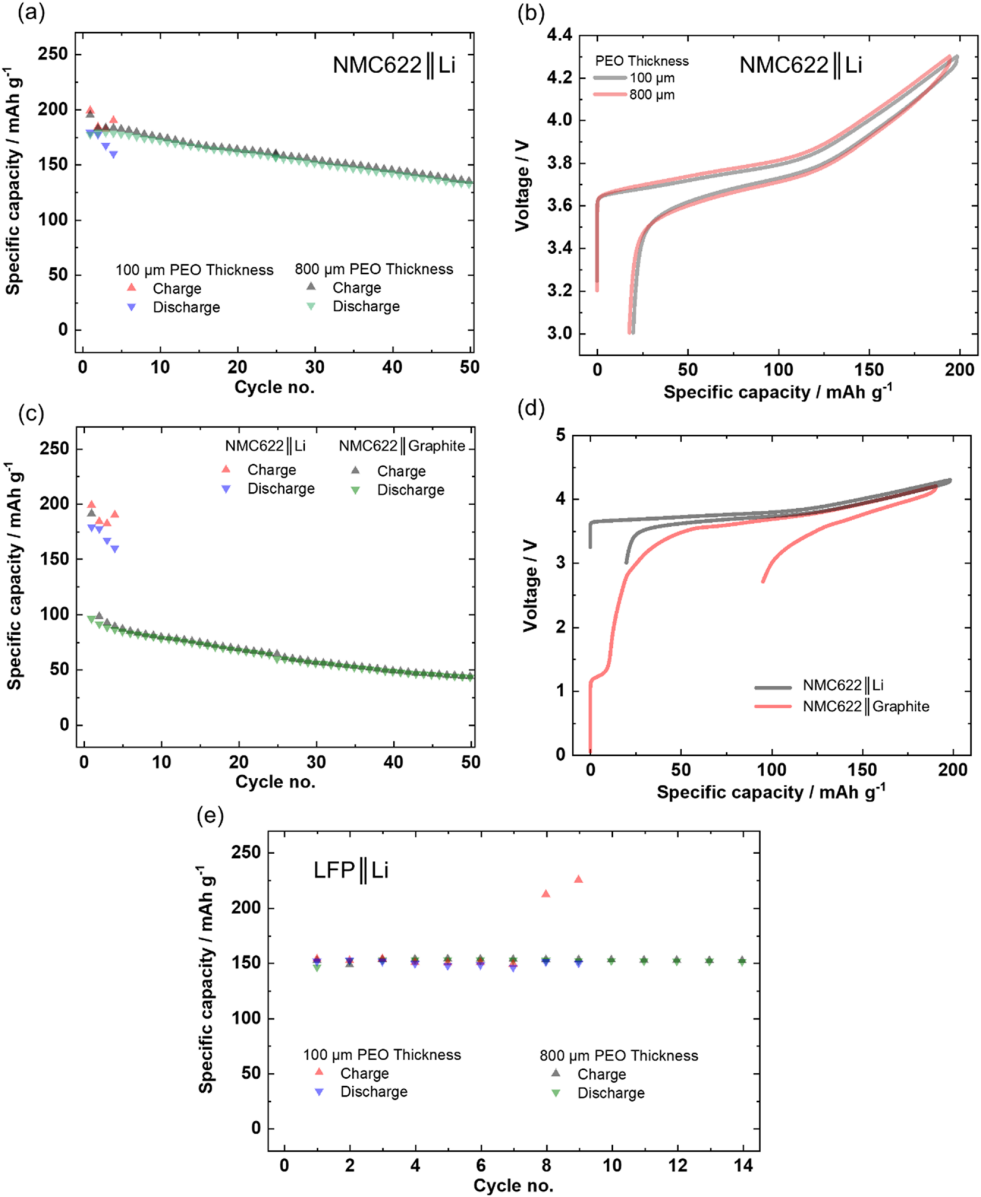
Galvanostatic charge/discharge cycling behavior with a specific current of 15 mA g^−1^ (≈0.1 C). (**a**) Increasing the thickness of the PEO based SPE (800 µm instead of 100 µm); demonstrates voltage noise-free performance with (**b**) similar voltage curves. (**c**) Varying the negative electrode (graphite instead of Li metal); demonstrate also voltage noise-free performance. (**d**) Voltage curves pointing at (compared to Li metal limited amount of transferable Li^+^) and SEI formation (=Li^+^ loss) as reason for decreased specific discharge capacity, when graphite is used. As in these experiments the SPE|NMC622 interface remained the same, experimental data point at the negative electrode and its interface to the SPE as source for voltage noise. (**e**) Voltage curves for the LFP||Li cell with varied SPE thickness validate that similar voltage noise failure can also occur in the well-known LFP-PEO-SPE system, finally pointing to the Li/SPE interface as the source of voltage noise.

The voltage noise failure due to dendrite penetration through the PEO-based SPE should be minimized when the thickness of the SPE membrane is increased. This can be reasoned by an increased Li dendrite path and/or by increased mechanical stability *e.g*. shear modulus^6^. The shear modulus can be regarded as a value for the ease of penetration through the material^46^. By increasing the shear modulus by e.g. increasing the membrane thickness, greater force is required to penetrate the material. Therefore, a charge/discharge cycling experiment is conducted where the thickness of PEO based SPE is increased *e.g*. eightfold to the value of ≈800 µm in assembled state (Fig. 4(a)).

A side note: The SPE thickness increase has a rather insignificant impact on the first charge/discharge cycle (Fig. 4(b)). Thus, the accompanied decrease in ionic conductance (depending on membrane thickness) is apparently insignificant for this experimental condition. Evaluation of the data in Fig. 4(a) reveals that voltage noise failure with a thin SPE membrane occurs rather early (here in the third cycle), as expected, while the cell with the thick SPE shows no failure in the first 50 charge/discharge cycles. This demonstrates that the SPE and the SPE|NMC622 interface is “working well” even during charge, again pointing at incompatibilities at the SPE|Li interface, such as dendritic micro-shorts originating from the Li metal electrode. The dependence on the membrane thickness is also validated for LiFePO_4_ (LFP) as positive electrode, as shown in Fig. 4(e). Independent of the positive electrode (thus of the positive electrode potential) the same behavior is obtained for LFP electrodes, namely a voltage noise-free performance for larger SPE thickness, while the cell with lower SPE thickness fails in the 8^th^ cycle. This example with a low voltage LFP electrode additionally demonstrates that the voltage noise failure of PEO-based SPEs is independent of electrode potentials at operation potentials below 4.6 V vs. Li|Li^+,^ but rather depends on Li dendrite penetration (higher mass loading (in the case 5–10 mg) results in more Li deposition and more dendrites during charge). Obviously, the origin of voltage noise failure arises from Li metal as negative electrode. Apparently, it is the Li dendrite penetrability of the SPE which is crucial for this failure, which should be modified and improved as an exemplary reasonable strategy, *e.g. via* cross linking^47^.

In the final experiment, the Li metal negative electrode is simply exchanged by a graphite resulting in a LIB cell configuration^48,49^. The observed lower specific capacities during charge/discharge cycling (Fig. 4(c)) can be attributed to the formation of a protecting solid electrolyte interphase (SEI)^50^ film on graphite, which is accompanied with irreversible Li consumption^51^, as indicated by a voltage plateau at ≈1.2 V (Fig. 4(d))^1,52,53^. Unlike Li metal based battery (LMB) cells, LIB cells with graphite electrodes cannot compensate this specific capacity loss, as there is no stoichiometric Li excess in the negative electrode^53^. Ether based electrolyte compounds are known to build ineffective SEI films on graphite, which may explain the relatively high specific capacity loss^1^. Nevertheless, the subsequent charge/discharge cycling proceeds without voltage noise failure. Because the SPE|NMC622 interface in the experiments with Li or graphite negative electrodes is the same, the different performance can be solely attributed to the different negative electrodes and the different anode interfaces to the SPE. One can conclude that the voltage noise failure of PEO-based SPE in a battery cell significantly depends on the used negative electrode and against intuition not on the positive electrode material and its operation potential.

## Conclusion

It is well-known, that poly(ethylene oxide) (PEO) based solid polymer electrolytes (SPEs) repeatedly fail in Li metal cells with high voltage positive electrodes, *e.g*. with LiNi_0.6_Mn_0.2_Co_0.2_O_2_ (NMC622), which is visible as noisy voltage/potential response during galvanostatic charge.

Simple, but so far not in publications described, diagnostic work was conducted to reveal the primary mechanism of voltage noise failure. The main oxidation onset of the PEO based SPE could be assigned to 4.6 V vs. Li|Li^+^ under galvanostatic and potentiodynamic conditions, which was checked for validation reasons on different active electrodes including different positive electrode structures and chemical compositions. The oxidation stability limit could be shown to be independent of molecular weight (MW) of the SPE in the investigated range between 0.3–5.0 m and salt concentration in the investigated range of (EO:Li) 12:1–20:1. The main oxidative decomposition reaction at the SPE|NMC622 seems not to be primarily relevant as possible source for the voltage noise failure.

Instead, we could attribute the voltage noise failure of these cells to reactions at the SPE|Li interface. In particular micro-shorts due to dendritic (high surface area) lithium (HSAL) formation during the charge process seem to be the responsible failure source. An increase in SPE thickness (longer distance between the electrodes and/or increased shear modulus) or a simple exchange of Li metal by graphite based negative electrode to counteract dendritic short-circuits, indeed revealed a charge/discharge cycling without the “voltage noise” failure.

This relation is also validated with LiFePO_4_ (LFP) based positive electrodes, which possess a lower upper cut-off potential than NMC based electrodes, which demonstrates that below 4.6 V vs. Li/Li^+^ not the type of active material and its operation potential at the positive electrode but rather the Li metal negative electrode behavior is responsible for the voltage noise failure.
